# Green Extraction of Polysaccharides from *Gleditsia japonica* Var. *delavayi* Seeds: Optimization and Physicochemical Properties

**DOI:** 10.3390/foods15071139

**Published:** 2026-03-26

**Authors:** Xiangzhong Mao, Chengyan Pi, Xiaowei Peng, Boxiao Wu, Changwei Cao, Huan Kan, Yun Liu, Fang Li

**Affiliations:** 1 College of Biological Science and Food Engineering, Southwest Forestry University, Kunming 650224, China; xiangzhong@swfu.edu.cn (X.M.); pichengyan1518739@163.com (C.P.); qqajx19@163.com (X.P.); wuboxiao@yafg.ac.cn (B.W.); ccwylf1111@swfu.edu.cn (C.C.); kanhuan@swfu.edu.cn (H.K.); 2 Forest Resources Exploitation and Utilization Engineering Research Center for Grand Health of Yunnan Provincial Universities, Southwest Forestry University, Kunming 650224, China

**Keywords:** *Gleditsia japonica* var. *delavayi*, polysaccharides, green extraction, optimization

## Abstract

The endosperm of *Gleditsia japonica* var. *delavayi* seeds is a valued medicinal and edible material, rich in polysaccharides exhibiting excellent functional properties for food applications. However, conventional methods for extracting *Gleditsia japonica* var. *delavayi* polysaccharides (GJP) are often inefficient and environmentally unfriendly. Thus, we developed a green, ultrasound-assisted process for extracting GJP. We systematically optimized key parameters (liquid-solid ratio and ultrasonic time, temperature, and power) using single-factor, Plackett–Burman and Box–Behnken experimental designs to maximize yield and characterize the product. The optimized process (200 mL/g, 62 min, 51 °C and 180 W) exhibited an extraction yield of 76.11%, producing GJP with a purity of 79.89%, which satisfies standards for food additives. The extracted GJP exhibited a semi-crystalline structure, high solubility (80.06%), low esterification degree (2.60%) and high viscosity and thermal stability between 30 °C and 70 °C. Crucially, this process required no chemical reagents and consumed only 0.18 kW·h of energy. Analysis indicates that the optimized ultrasound-assisted extraction of GJP is a green and efficient method with high extraction rates and reduced processing time and energy consumption; furthermore, it does not require any chemical reagents, making it a promising alternative to conventional techniques.

## 1. Introduction

*Gleditsia japonica* var. *delavayi* is a plant belonging to the genus *Gleditsia* within the Fabaceae family, with the endosperm of its seeds being a medicinal and edible substance [1]. The endosperm of *G. japonica* var. *delavayi* seeds primarily comprises polysaccharides, proteins, and minerals. The polysaccharides are the most abundant component, accounting for 50–80% of the seed mass, whereas the protein content is generally 2.37–3.64% [1,2]. Additionally, owing to their excellent thickening, emulsifying, and stabilizing properties, polysaccharides are widely used as functional ingredients in food additives [3,4]. Currently, extraction and purification serve as the primary methods for obtaining high-purity polysaccharides. However, conventional processes for extracting polysaccharides often suffer from limitations such as complex procedures, low efficiency, and environmental concerns, which hinder their broader application [5].

A variety of methods have been employed for extracting polysaccharides from plant materials. Traditional extraction methods include hot-water extraction [6], acid extraction [7], and alkali extraction [8]. Despite their simplicity, traditional extraction methods typically exhibit inherently low extraction efficiency and product purity. To overcome these limitations, new extraction techniques have been developed, including ultrasound-assisted extraction [9], supercritical fluid extraction [10] and microwave-assisted extraction [11]. Ultrasonic technology has been widely adopted for polysaccharide extraction in recent years because of its high efficiency, cost-effectiveness and environmental friendliness. Ye et al. [12] reported that the ultrasound-assisted extraction of polysaccharides from *Rubia cordifolia* L. waste stems increased the yield by 77.2% and reduced the extraction time by 76% compared to traditional hot-water extraction. Therefore, herein, ultrasound-assisted extraction was employed to separate *G. japonica* var. *delavayi* polysaccharides (GJP) from the endosperm.

Furthermore, a number of factors in the ultrasound-assisted extraction affect polysaccharide extraction efficiency, most notably liquid-solid ratio, number of extractions and ultrasonic time, temperature and power [13,14]. Chen et al. [15] investigated the effects of ultrasonic power and ultrasonic time on the yield and physicochemical properties of *Flammulina velutipes* polysaccharides. Glaue et al. [16] studied the effects of liquid-solid ratio and ultrasonic time on the extraction of okra polysaccharides. These studies primarily evaluated the effects of individual parameters on the extraction yield, without a comprehensive assessment of their interactions. Furthermore, the combined effects of these factors on the physicochemical properties of polysaccharides have not been elucidated. Therefore, herein, the liquid-solid ratio, number of extractions and ultrasonic time, temperature and power were systematically optimized, and the physicochemical properties of the extracted polysaccharides were determined.

Moreover, full-factorial experiments can yield comprehensive results, but it often requires a significant investment of time and labor in experiments involving multiple factors and levels. Appropriate experimental designs, including Plackett–Burman, steepest-climb experiment and response-surface methodology, offer efficient alternatives. They enable efficient identification of key variables and elucidation of their interactions through simultaneous variation in multiple factors [17]. Among these, Plackett–Burman design serves as an efficient screening method to rapidly identify key factors with significant effects from numerous factors [18,19]. Subsequently, a steepest-climb experiment was employed to determine the optimization direction and optimal response region for these key factors. Concurrently, to gain insight into factors interactions, it is also necessary to associate factors and response value utilizing response-surface methodology [20]. Among the various RSM designs, the Box–Behnken design requires fewer experimental runs, avoids extreme factor combinations, and accurately captures interactions between factors [18,19,20]. Therefore, this study employed a combined Plackett–Burman and Box–Behnken approach to determine the optimal process parameters for GJP extraction.

Research on GJP remains in its infancy, with existing extraction methods plagued by cumbersome processes, low efficiency, and high energy consumption. Thus, the present study proposes a green, efficient, and environmentally friendly extraction technique, which is a promising alternative to conventional methods. We optimized the parameters of the ultrasound-assisted extraction of GJP using Plackett–Burman (PB) and Box–Behnken (BB) experimental designs. Additionally, the solubility, viscosity, esterification degree and crystal structure of the extracted GJP were analyzed to assess its application potential in the food industry. The results of the present study are expected to provide data support and a theoretical basis for the green extraction, development and utilization of GJP.

## 2. Materials and Methods

### 2.1. Materials

The endosperm of *G. japonica* var. *delavayi* seed, collected from Lianghe, Yunnan Province, China, was pulverized in a mill (YB-250A, Sufeng Industry and Trade Co., Ltd., Suichang, China) and passed through a 60-mesh sieve (pore diameter 0.25 mm). The powder was dried for 3 h in a heat pump dryer (DNF-4K, Southeast Wind Power Technology Co., Ltd., Beijing, China) at 40 °C, resulting in a final moisture content of approximately 3–6%. The dried powder was stored in a desiccator for subsequent use.

### 2.2. Reagents

The reagents used in this study including phenol (C_6_H_6_O, purity ≥99%, Shandian Pharmaceutical Co., Ltd., Kunming, China), sodium hydroxide (NaOH, purity ≥96%, Chengdu Jinshan Chemical Reagent Co., Ltd., Chengdu, China), glucose (C_6_H_12_O_6,_ purity ≥99%, Tianjin Damao Chemical Reagent Factory Co., Ltd., Tianjin, China), phenolphthalein (C_20_H_14_O_4_, purity ≥99%, Tianjin Fengchuan Chemical Reagent Technology Co., Ltd., Tianjin, China), anhydrous ethanol (C_2_H_5_OH, purity ≥99%, Tianjin Fengchuan Chemical Reagent Technology Co., Ltd., Tianjin, China), potassium bromide (KBr, purity ≥99%, Tianjin Kemiou Chemical Reagent Co., Ltd., Tianjin, China), sulfuric acid (H_2_SO_4_, purity 95–98%, Yunnan Yanglin Industrial Development Zone Youdian Pharmaceutical Co., Ltd., Kunming, China), hydrochloric acid (HCl, purity 36–38%, Yunnan Yanglin Industrial Development Zone Youdian Pharmaceutical Co., Ltd., Kunming, China), Coomassie Brilliant Blue G-250 (C_47_H_48_N_3_NaO_7_S_2_, purity ≥95%, Sygenta Biotech GmbH, Maintal, Germany).

### 2.3. Optimization of Extraction Conditions

The present study employed a sequential experimental design strategy to systematically optimize the parameters of the ultrasound-assisted extraction of GJP, aiming to maximize yield. The optimization process comprised four sequential stages. First, single-factor experiments were conducted to preliminarily assess the effect trends of each variable and determine their minimum and maximum values. Subsequently, a PB design was employed for rapid screening to identify key factors that significantly affect extraction yield. Building on this foundation, steepest-climb experiments were conducted to approach the optimal response region along the gradient, performing range screening on the selected key variables. Finally, response-surface analysis was employed to establish interaction models amongst key factors and determine the optimal extraction conditions.

#### 2.3.1. Single-Factor Experiments

Based on relevant literature, particularly the research by Liu et al. [1], the initial range and center value for the single-factor experiments were determined. Table 1 shows the experimental design of the single-factor experiment. Experiments 1–5 investigated the effect of liquid-solid ratio on the GJP extraction yield. Experiments 6–10, 11–15, and 16–20 were conducted to evaluate the effects of ultrasonic time, ultrasonic temperature, ultrasonic power on GJP extraction yield, respectively. Experiments 21–25 assessed the impact of the number of extractions on the GJP extraction yield.

#### 2.3.2. Plackett–Burman Experiments

The PB design was employed to screen key factors affecting ultrasound-assisted GJP extraction. The liquid-solid ratio (*X*_1_), ultrasonic time (*X*_2_), ultrasonic temperature (*X*_3_), ultrasonic power (*X*_4_), and the number of extractions (*X*_5_) were selected as experimental factors, with their respective ranges determined through preliminary experiments (Supplementary Table S1). Based on a two-level (high and low) experimental design, a standard Plackett–Burman design matrix for 12 runs was constructed to conduct extraction experiments at the high (+1) or low (−1) levels of each factor. The experimental sequence was randomized to minimize systematic bias. Subsequently, a first-order polynomial model based on Equation (1) was employed to construct the PB model, aiming to identify the key variables affecting the extraction process [21,22].

(1) $$Y=\lambda_{0}+\sum_{i=1}^{5}\lambda_{i}x_{i}$$

where *Y* is the observed response value (GJP yield), *λ*_0_ is the model intercept, *λ_i_* is the regression coefficient and *x_i_* is an independent variable.

#### 2.3.3. Steepest-Climb Experiments

Based on the results of PB experiments, three factors, namely, liquid-solid ratio (*X*_1_), ultrasonic time (*X*_2_) and ultrasonic temperature (*X*_3_), were identified to have the most significant effects on the GJP yield and were therefore selected for further optimization. Although ultrasonic power (*X*_4_) and the number of extraction cycles (*X*_5_) also showed statistically significant effects, they were fixed in subsequent experiments based on the results of preliminary single-factor tests. In particular, ultrasonic power was set to 180 W, as higher power resulted in only marginal yield improvements while substantially increasing energy consumption, which conflicts with green extraction principles. The number of extraction cycles was fixed at one, as additional cycles did not significantly enhance extraction efficiency but increased solvent use and processing time.

To rapidly approach the region of maximum response, a steepest-climb experiment was conducted along the gradient defined by the three key factors (*X*_1_, *X*_2_ and *X*_3_). The step size for each factor was determined based on the regression coefficients obtained from the Plackett–Burman model and was proportional to the magnitude of each factor’s effect. Specifically, since the coefficient for ultrasonic temperature (*X*_3_) was the largest, its step size of 5 °C was set as the reference value. The step sizes for the liquid-to-solid ratio (*X*_1_) and ultrasonication time (*X*_2_) were scaled accordingly and, without significantly deviating from the gradient direction, adjusted to 25 mL/g and 5 min, respectively, to facilitate experimental operation. The levels for each factor in this experiment are listed in Supplementary Table S2. The optimal conditions identified from the steepest-climb path were then used as the central point in the subsequent response-surface optimization.

#### 2.3.4. Box–Behnken Experiments

After identifying key factors and their approximate optimal regions through PB and steepest-climb experiments, BB design experiments with three factors at three levels were employed to model and optimize the interactions amongst the liquid-solid ratio (*X*_1_), ultrasonic time (*X*_2_) and ultrasonic temperature (*X*_3_). A total of 17 experiments were conducted (Table 2). A second-order polynomial model was fitted to relate each parameter to the response [21,22]:

(2) $$Y=\lambda_{0}+\sum_{i=1}^{3}\lambda_{i}x_{i}+\sum_{i=1}^{3}\lambda_{ii}{x_{i}}^{2}+\sum_{i=1}^{3}\times \sum_{j=i+1}^{3}\lambda_{ij}x_{i}x_{j}$$

where *Y* is the predicted response value, *λ*_0_ is the intercept, *λ_i_* is the regression coefficient, *λ_ii_* is the second-order regression coefficient, *λ_ij_* is the interaction regression coefficient and *x_i_* and *x_j_* are independent variables.

### 2.4. Ultrasonic-Assisted GJP Extraction

Sample powder (0.1 g) was weighed and placed in a 50 mL centrifuge tube. Distilled water was added to achieve the designed liquid-solid ratio. The sealed centrifuge tube was then fully immersed and secured at the center of an ultrasonic cleaning bath (SB-500DTY, Xinzhi Ultrasonic Equipment Co., Ltd., Ningbo, China). This device operates at an ultrasonic frequency of 33 kHz, with an ultrasonic power of up to 500 W and a heating power of 1000 W. Given the internal dimensions of the tank (500 × 300 × 150 mm^3^), the calculated average ultrasonic intensity of the system is 0.33 W/cm^2^, determined as the ultrasonic power divided by the base area of the tank. Extraction was performed at set ultrasonic time, temperature, and power levels. The ultrasonic temperature was precisely controlled using a digital heating system built in the bath, which maintained the temperature at the target value with an accuracy of ±1 °C. Throughout extraction, the centrifuge tubes were manually inverted three times every 10 min to ensure uniformity and prevent sedimentation. After process completion, the suspension was centrifuged at 2570 *g* and 25 °C for 10 min. The supernatant was collected and mixed with anhydrous ethanol at a 1:4 (*v*/*v*) ratio. Then, the mixture was allowed to stand at 4 °C for 12 h to facilitate precipitation, after which the precipitate was collected through centrifugation at 2570 *g* and 25 °C for 10 min. Subsequently, the precipitate was dissolved in 50 mL of distilled water, and the solution was directly used to determine the GJP concentration.

**Figure 1:**
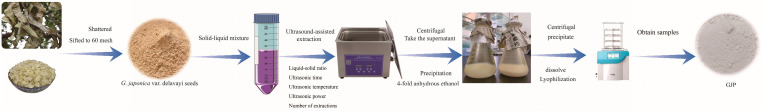
Schematic diagram of ultrasonic-assisted extraction process of *Gleditsia japonica* var. *delavayi* polysaccharides (GJP).

Additionally, the GJP solution obtained under optimal ultrasonication conditions was spread over the bottom of 10.5 × 1.9 cm^2^ culture dishes, filling each dish halfway, and pre-frozen at −80 °C for 3 h. The frozen liquid was freeze-dried using a vacuum freeze dryer (FD5-3, SIM International Group Co., Ltd., Newark, NJ, USA) at −60 °C for 48 h, yielding GJP [23]. The GJP extraction process is shown in Figure 1.

### 2.5. Determination of the GJP Concentration

The GJP concentration was evaluated through the phenol–sulfuric acid method [24]. Specifically, 1 mL of a GJP solution obtained as described in Section 2.4, 1 mL of a 5% (*w*/*v*) phenol solution, and 5 mL of sulfuric acid were mixed in a 25 mL test tube. The mixture was left to stand at 25 °C for 10 min and then incubated in a water bath (YRE-2000E, Yuhua Yiqi Co., Ltd., Gongyi, China) at 30 °C for 20 min. Subsequently, the absorbance of the mixture was measured using ultraviolet (UV) spectrophotometry (UV-2600, Shimadzu Instruments Co., Ltd., Ningbo, China). The detection wavelength was 490 nm, and the optical path of the quartz cuvette was 10 mm. Glucose was used as the calibration standard.

The GJP extraction yield (*Y*) was calculated using Equation (3).

(3) $$Y(\%)=\frac{C\times V\times F}{m}\times 100$$

where *C* is the GJP concentration (mg/mL), *V* is the total volume of the GJP solution (mL), *F* is the dilution factor and *m* is the mass of the powdered *G. japonica* var. *delavayi* endosperm (in mg; 100 mg herein).

### 2.6. Energy Consumption

Following the method of Khai et al. [25], the energy consumption of ultrasound-assisted extraction was calculated using Equation (4).

(4) Q(kW⋅h)=P×t

where *Q* is the energy consumed (kW·h), *P* is the ultrasonic power (W) and *t* is the ultrasonic time (h).

### 2.7. Physicochemical Properties of GJP

#### 2.7.1. Purity of GJP

Crude, freeze-dried GJP powder (4 mg) was dissolved in 40 mL of distilled water and topped to 50 mL in a volumetric flask to prepare a test solution. The concentration of polysaccharides in this solution was determined using the phenol–sulfuric acid method described in Section 2.5. The purity of GJP (*P*) was calculated using Equation (5).

(5) $$P(\%)=\frac{C_{1}\times V_{1}}{m_{1}}\times 100$$

where *C*_1_ is the polysaccharide concentration in the test solution (mg/mL), *V*_1_ is the total volume of the test solution (in mL; 50 mL herein) and *m*_1_ is the mass of the crude, freeze-dried GJP powder used to prepare the test solution (4 mg).

#### 2.7.2. Determination of Protein Content

The protein content of GJP was determined according to the method of Barbosa et al. [26] with slight modifications. Briefly, 4 mg of GJP was mixed with 4 mL of distilled water in a 15 mL centrifuge tube. Subsequently, 1 mL of the resulting GJP solution was combined with 5 mL of a 0.02 g/L Coomassie Brilliant Blue G-250 solution and kept in the dark for 5 min. The absorbance of the mixture was measured in a quartz cuvette using UV spectrophotometry (UV-2600, Shimadzu Instruments Co., Ltd., Ningbo, China). The detection wavelength was set to 595 nm, and bovine serum albumin served as the calibration standard. The protein content of GJP (*PC*) was calculated using Equation (6).

(6) $$PC(\%)=\frac{c\times v\times N}{m}\times 100$$

where *c* is the protein content of the GJP solution, mg/mL; *v* is the volume of the GJP solution, mL; *N* is the dilution factor of the GJP solution; and *m* is the GJP mass, mg.

#### 2.7.3. Determination of GJP Solubility

The solubility of GJP was determined according to the method of Xu et al. [27] with minor modifications. Specifically, 5 mg of GJP and 5 mL of distilled water were added to a 50 mL centrifuge tube. The suspension was processed using a vortexer (Joanlab VM-300, Qun’an Scientific Instruments Co., Ltd., Huzhou, China) for 30 s. Subsequently, the suspension was centrifuged at 7150 *g* and 25 °C for 10 min, after which the supernatant was discarded. The centrifuge tubes with precipitate were placed in a hot air dryer (DHG-9240A, Qixin Scientific Instruments Co., Ltd., Shanghai, China) at 55 °C for 24 h. The solubility of GJP (*S*) was evaluated using Equation (7).

(7) $$S(\%)=\frac{m_{0}-m_{1}}{m_{0}}\times 100$$

where *m*_0_ is the mass of GJP and the centrifuge tube (mg) and *m*_1_ is the mass of the dried precipitate and the centrifuge tube (mg).

#### 2.7.4. Determination of GJP Viscosity

GJP (1.00 g) was dissolved in 150 mL of distilled water. The GJP solution was placed in a water bath (XMTD-7000, Yongguangming Medical Instruments Co., Ltd., Beijing, China) and equilibrated at 30 °C, 50 °C or 70 °C for 15 min. Then, the viscosity of the GJP solution was determined using a rotational viscometer (Brookfield KU-3, Shanghai Hecha Industrial Co., Ltd., Shanghai, China) with a KU-1030 rotor at a speed of 60 rpm [28].

#### 2.7.5. GJP Esterification Degree (Ed)

The esterification degree of GJP was determined using the titration method described by Lira-Ortiz et al. [29]. Specifically, 200 mg of GJP was dissolved in distilled water and diluted to the mark in a 50 mL volumetric flask. Two drops of phenolphthalein indicator were added to the prepared GJP solution, followed by titration with 0.1 mol/L NaOH (*V*_1_). Then, 10 mL of 0.1 mol/L NaOH was added to the titrated solution, followed by thorough mixing and 15 min of standing. Next, 10 mL of 0.1 mol/L HCl was added to the solution, followed by titration with 0.1 mol/L NaOH (*V*_2_). The esterification degree of GJP (*ED*) was calculated using Equation (8):

(8) $$ED(\%)=\frac{V_{1}}{V_{1}+V_{2}}\times 100$$

where *V*_1_ is the volume of NaOH consumed in the titration of a 50 mL GJP solution (mL) and *V*_2_ is the volume of NaOH consumed in the titration of esterified carboxyl groups in the sample solution (mL).

### 2.8. Characterization of GJP Structure

#### 2.8.1. Fourier Transform Infrared (FTIR) Spectroscopy

FTIR spectroscopy (FT-IR650, Gangdong Technology Co., Ltd., Tianjin, China) was employed to identify the functional groups in GJP. A mixture of 2 mg GJP and 200 mg KBr was ground into a homogeneous powder in an agate mortar and pressed into a pellet. The spectrum was acquired by averaging 32 scans at a resolution of 4 cm^−1^ over a wavenumber range of 4000–500 cm^−1^ [30]. The raw data were then exported and further processed using Origin 2021 (Origin, Northampton, MA, USA).

#### 2.8.2. X-Ray Diffraction (XRD) Characterization

The crystal structure of GJP was determined using XRD (Ultima IV, Nippon Rigaku Co., Ltd., Akishima, Japan) with Cu Kα radiation. The GJP powder was loaded onto a glass slide, ensuring a level surface. The 2*θ* scan range was 5–70°, and the scanning speed was 5°/min [31].

### 2.9. Statistical Analysis

Design Expert 12 (Design Expert, Minneapolis, MN, USA) was used for constructing experimental designs, including PB, steepest-climb and response-surface experiments. The statistical significance of differences was determined by one-way analysis of variance (ANOVA) implemented in SPSS 26.0 (SPSS, Chicago, IL, USA). Statistical significance was defined as *p* < 0.05. The results were expressed as mean ± standard deviation. The graphs were constructed using Origin 2021 (Origin, Northampton, MA, USA). All experiments were conducted in triplicate.

## 3. Results and Discussion

### 3.1. Single-Factor Experiments on Ultrasound-Assisted GJP Extraction

**Figure 2:**
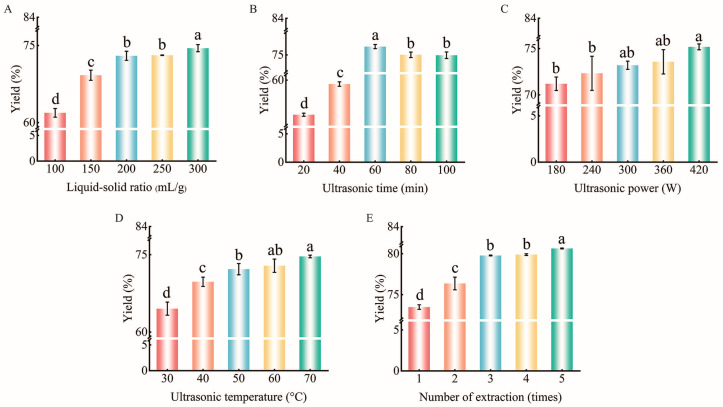
The influence of liquid-solid ratio (**A**), ultrasonic time (**B**), ultrasonic power (**C**), ultrasonic temperature (**D**), number of extractions (**E**) on GJP extraction yield. The different letters on the bar indicate significant differences (*p* < 0.05) at different extraction parameters.

Figure 2A–E show the effects of liquid-solid ratio, ultrasonic time, ultrasonic power, ultrasonic temperature, and the number of extractions on the GJP extraction yield, respectively. The extraction yield of GJP shows an upward trend with increasing liquid-solid ratio. As the liquid-solid ratio increased from 100 to 200 mL/g, the extraction yield of GJP rose from 61.90% ± 0.84% to 72.98% ± 0.88% (*p* < 0.05). When the liquid-solid ratio was further increased to 250 and 300 mL/g, the extraction yield of GJP plateaued, stabilizing within an equilibrium range from 73.11% ± 0.13% to 74.46% ± 0.69% (Figure 2A). The subsequent single-factor experiments were conducted at a liquid-solid ratio of 200 mL/g based on the critical points of extraction yield. The initial increase in the GJP extraction yield (liquid-solid ratio < 200 mL/g) is likely caused by improved mass transfer and solubility, driven by a higher concentration gradient [32]. The plateau at 200 mL/g suggests that the extraction system reached a dissolution equilibrium, where solvent availability was no longer the limiting factor [33,34,35]. Thus, the extraction yield of GJP may be governed by the number of extractions and ultrasonication time, temperature and power.

Figure 2B shows the effect of ultrasonic time on the GJP extraction yield. With the extension of ultrasonic time from 20 to 60 min, the extraction yield of GJP increased from 53.91% ± 0.27% to 76.5% ± 0.39%. However, with further extensions to 80 and 100 min, the extraction yield declined to 75.02% ± 0.48% and 74.87% ± 0.56%, respectively. This decline was attributed to the degradation of GJP caused by the excessive thermal and mechanical effects of ultrasound [36,37].

As shown in Figure 2C, the extraction yield of GJP increased with ultrasonic power. With an increase in ultrasonic power from 180 to 420 W, the GJP extraction yield increased from 71.17% ± 0.72% to 75.23% ± 0.34%. Gao et al. [38] investigated the ultrasonic extraction of total flavonoids from mung beans, reporting that the flavonoid extraction rate remained relatively stable across ultrasonic power levels from 100 to 500 W, fluctuating between 1.94 and 2.22 mg/g, respectively. This observation is consistent with the results of the present study. Herein, with an increase in ultrasonic power from 180 to 420 W, the extraction yield of GJP rose by 4.0%, but the energy consumption increased from 0.18 to 0.42 kW·h, respectively. Based on the comprehensive consideration of extraction yield and economic viability, the ultrasonic power of 180 W was selected for the subsequent single-factor experiments.

Figure 2D shows the effect of ultrasonic temperature on the GJP extraction yield. As the ultrasonic temperature was increased from 30 °C to 70 °C, the extraction yield of GJP rose from 64.53% ± 1.27% to 74.66% ± 0.26% (*p* < 0.05). The significant effect of temperature on GJP extraction yield was possibly due to the softening of plant cell walls at high temperatures, which reduced the release resistance of GJP [39]. Moreover, high temperature imparted thermal kinetic energy to GJP molecules, which enhanced the probability of GJP molecules diffusing away from the solid matrix [40]. These results indicated that during ultrasound-assisted extraction, the process shifted from being kinetically controlled to thermodynamically controlled.

As shown in Figure 2E, the number of extractions significantly affected the extraction yield of GJP. An increase in the number of extractions from one to three resulted in the rise in the extraction yield of GJP from 73.48% ± 0.29% to 80.64% ± 0.07%, an increase of 7.16%. However, with a further increase in the number of extractions to three and five times, the extraction yield of GJP did not significantly change, remaining at ~80.0%.

### 3.2. Plackett–Burman and Steepest-Climb Experiments for Ultrasonic-Assisted Extraction of GJP

PB experiments employ a two-level experimental design with low and high levels. A comparison of differences between the two levels and the overall difference is used to determine the significance of factor effects. This factor screening aims to conserve experimental materials and enhance the experimental response value. Based on the trends observed in the single-factor experiments, a PB design was employed to statistically determine which of the five factors (*X*_1_–*X*_5_) exerted a significant effect on the GJP extraction rate. The design matrix and corresponding extraction rates are detailed in Supplementary Table S3. The ANOVA results for the PB model are presented in Table 3.

Table 3 shows that the model *F*-value is 15.84 (*p* = 0.0021), indicating statistical significance. The *R*^2^ value of 0.9296 suggests that 92.96% of the experimental data can be explained by this model. The adjusted *R*^2^ (*R*^2^Adj) of 0.8709 is highly consistent with *R*^2^, confirming the high predictive accuracy of the model. The effects of factors significantly affecting GJP yield decrease in the following order: *X*_3_ > *X*_2_ > *X*_1_ > *X*_5_ > *X*_4_. Based on experimental results and practical application, three factors (liquid-solid ratio, ultrasonic time, and ultrasonic temperature) were selected for a BB design.

Supplementary Table S4 shows the design and results of the steepest-climb experiment. By simultaneously increasing the liquid-solid ratio, ultrasonic time and ultrasonic temperature along a preset path, the synergistic effects of these three factors on GJP extraction yield were investigated. The extraction yield peaked at approximately 200 mL/g, 60 min, and 50 °C (76.11%), suggesting optimal synergy amongst mechanical disruption, thermal dissolution, and mass transfer processes under these conditions and indicating that the extraction threshold had been reached. Beyond this point, yield declined, suggesting that excessively prolonged or intense processing may induce polysaccharide degradation or side reactions. Thus, the steepest-ascent experiment successfully identified the optimal parameter region, providing a precise optimization center for subsequent response-surface experiments.

### 3.3. Analysis of the Box–Behnken Experiment

#### 3.3.1. Design of Box–Behnken Experiments and Anova of the GJP Extraction Yield

The design of the BB experiment and the extraction yield of GJP are listed in Supplementary Table S5. The predicted responses were calculated using the second-order polynomial model as follows:

*Y* = 75.20 + 0.4875*X*_1_ + 1.4*X*_2_ + 1.46*X*_3_ − 0.0275*X*_1_*X*_2_ + 0.8675*X*_1_*X*_3_ − 1.1*X*_2_*X*_3_ − 1.71*X*_1_^2^ − 2.13*X*_2_^2^ − 2.27*X*_3_^2^.

ANOVA results for the experimental model are presented in Table 4. The model was statistically significant (*F*-value = 49.26 and *p*-value = 0.0001) and showed a good fit, as evidenced by a nonsignificant lack-of-fit (*F*-value = 1.65 and *p*-value = 0.3136) and a high coefficient of determination (*R*^2^ = 0.9845) [41]. The model exhibited high predictive accuracy, which was further confirmed by the close agreement between the *R^2^* and adjusted *R*^2^ (*R*^2^_Adj_ = 0.9645). Therefore, the model can be used for the analysis and prediction of the ultrasound-assisted extraction yield of GJP.

Regression analysis indicated a significant nonlinear correlation of *X*_2_ and *X*_3_ with the GJP extraction yield. The linear terms (*X*_2_ and *X*_3_), the *X*_2_*X*_3_ interaction, and the quadratic terms (*X*_1_^2^, *X*_2_^2^, and *X*_3_^2^) all had *p*-values below 0.05, suggesting their significant effects on the GJP extraction yield. Although the *X*_1_*X*_2_ interaction term was not statistically significant (*p* > 0.05), it was retained in the final model equation to preserve the complete hierarchical structure of the quadratic model corresponding to the BB design and provide full transparency regarding the initial software output. At the same time, factor significance analysis revealed that ultrasonication parameters markedly affected the GJP extraction yield. Ultrasonic temperature (*X*_3_) exerted the greatest effect, followed by ultrasonic time (*X*_2_) and the liquid-solid ratio (*X*_1_) [42].

#### 3.3.2. Response-Surface Analysis of the GJP Extraction Yield

**Figure 3:**
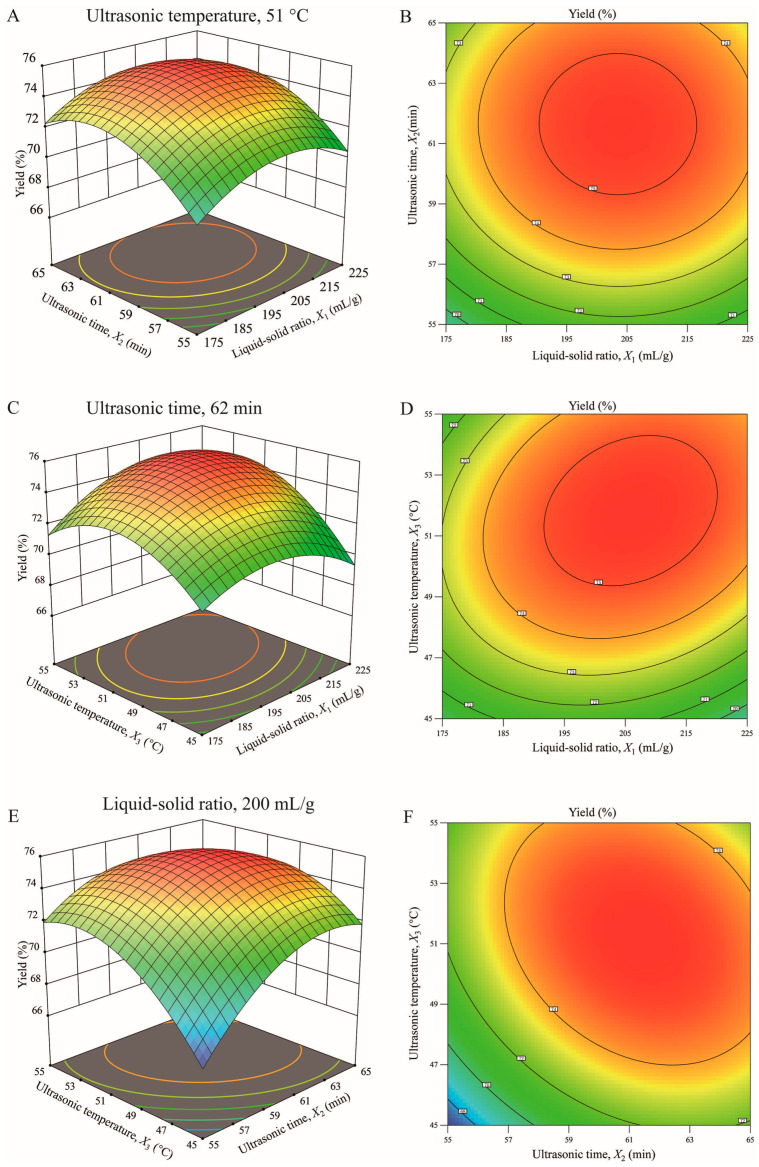
3-D response-surface plots and 2-D contour plots showing effects of various parameters on extraction yield of GJP. (**A**): The interactive effect of liquid-solid ratio *X*_1_ and ultrasonic time *X*_2_ on yield; (**B**): The contour plot of yield as a function of liquid-solid ratio *X*_1_ and ultrasonic time *X*_2_; (**C**): The interactive effect of liquid-solid ratio *X*_1_ and ultrasonic temperature *X*_3_ on yield; (**D**): The contour plot of yield as a function of liquid-solid ratio *X*_1_ and ultrasonic temperature *X*_3_; (**E**): The interactive effect of ultrasonic temperature *X*_3_ and ultrasonic time *X*_2_ on yield; (**F**): The contour plot of yield as a function of ultrasonic temperature *X*_3_ and ultrasonic time *X*_2_. The colors range from light to dark, representing a trend from low to high yield.

To further elucidate the interactions between different factors, a response-surface plot was constructed (Figure 3). Contour plots and three-dimensional (3D) graphs can effectively illustrate the strength and significance of interactions. The stronger the interaction between the two factors, the steeper the response surface and the more elliptical the contour plot [43]. Specifically, the contour plot of the interaction between liquid-solid ratio (*X*_1_) and ultrasonic time (*X*_2_) (Figure 3B) exhibits an approximately circular shape, with its corresponding 3D surface (Figure 3A) being relatively flat and symmetrical. This indicates that the interaction between the two factors is not statistically significant (*p* = 0.9119; Table 4). In stark contrast, the contour plots for the interactions involving ultrasonic temperature (*X*_3_) exhibit a distinct elliptical shape. The interaction plot for liquid-solid ratio and ultrasonic temperature (*X*_1_*X*_3_; Figure 3D) has an elliptical shape, whereas the 3D surface (Figure 3C) appears steep. This perfectly aligns with the significant interaction term for *X*_1_*X*_3_ (*p* = 0.0085). Furthermore, the interaction between ultrasonication time and temperature (*X*_2_*X*_3_; Figure 3F) exhibits the most pronounced elliptical contour, with its 3D surface (Figure 3E) having the steepest gradient. This provides strong evidence for the highly significant interaction identified in the ANOVA (*p* = 0.0025; Table 4).

#### 3.3.3. Identification and Verification of Optimal Extraction Conditions

Response-surface methodology was applied to fit the experimental data via multivariate regression, enabling the determination of optimal conditions through the analysis and visualization of 3D response-surface plots. The optimal parameters of GJP extraction were a liquid-solid ratio of 200 mL/g, an ultrasonic time of 61.4 min, an ultrasonic temperature of 50.5 °C, an ultrasonic power of 180 W, and one extraction cycle. The predicted extraction yield of GJP was 75.48%. Owing to practical constraints, the theoretical parameters were modified to a liquid-solid ratio of 200 mL/g, an ultrasonic time of 62 min, an ultrasonic temperature of 51 °C, an ultrasonic power of 180 W, and a single number of extractions for the actual experiments. Under practical conditions, the extraction yield of GJP was determined to be 76.11% ± 0.43% (Supplementary Table S6). The average relative error of the model prediction was 0.83%, and the relative standard deviation of the validation experiments was only 0.43%. Both error metrics are well below the empirical threshold of 10%, indicating that the established response-surface model is highly reliable and consistent.

**Figure 4:**
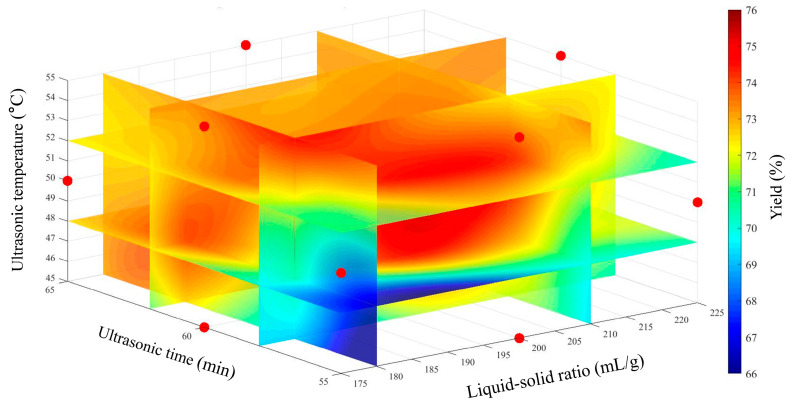
The 4D interaction graph of extraction parameters on GJP extraction efficiency. The red dots in the figure represent experimental data markers. The red dots represent the actual experimental points in the Box-Behnken design.

Furthermore, to analyze the effects of liquid-solid ratio, ultrasonic time and ultrasonic temperature on the extraction yield of GJP, a four-dimensional (4D) graph of the extraction yield as a function of independent variables was generated using the MATLAB 2024 slice function (Figure 4). The program is provided in Supplementary Text S1. Color change intuitively indicates the effects of the liquid-solid ratio, ultrasonic time and ultrasonic temperature on the extraction yield of GJP. As the liquid-solid ratio was increased, the GJP extraction yield increased. Based on the color change along the *Y*-axis, the extraction yield of GJP tended to increase with ultrasonic temperature. Importantly, the reddest region at the center of the graph indicates the parameter combination yielding the optimal extraction yield, which perfectly aligns with the predictions from the response-surface analysis. Thus, this visualization can be employed to preliminarily predict the parameter combinations required to achieve a specific extraction yield.

### 3.4. Purity and Protein Content of GJP

As shown in Table 5, the ultrasound-assisted extraction not only achieved a GJP extraction yield of 76.11% but also produced GJP with a purity of 79.89%. Remarkably, the protein content of GJP was merely 0.18% without implementing any deproteinization procedure. This indicates that the extraction method and optimal parameters can effectively prevent the dissolution and contamination of large amounts of proteins while yielding high-purity GJP. Therefore, the proposed process eliminated the necessity for further GJP purification, preventing the potential loss of polysaccharides and the generation of environmental pollution associated with traditional deproteinization processes.

Furthermore, according to the national standard GB 1886.322-2021 [44], polysaccharides with purity exceeding 60% can be used as food additives. The GJP obtained herein under optimal extraction parameters meets the requirements for food additives.

### 3.5. Solubility of GJP

Polysaccharides with high solubility are readily absorbed and utilized by living organisms [45]. As shown in Table 5, the solubility of ultrasonically extracted GJP was 80.06% ± 2.45%. Rasoul et al. [46] reported that the solubility of galactomannan isolated from *Gleditsia capsica* (Persian honey locust) seeds ranged between 63.90% and 65.30%, whereas Liu et al. [47] found that the solubility of galactomannans extracted from seeds of *Gleditsia sinensis* Lam was 70.11%. These values are markedly lower than the solubility achieved herein. Thus, GJP obtained via our optimized extraction method exhibits excellent solubility, highlighting its strong potential for application in polysaccharide-based products.

### 3.6. Viscosity of GJP

**Figure 5:**
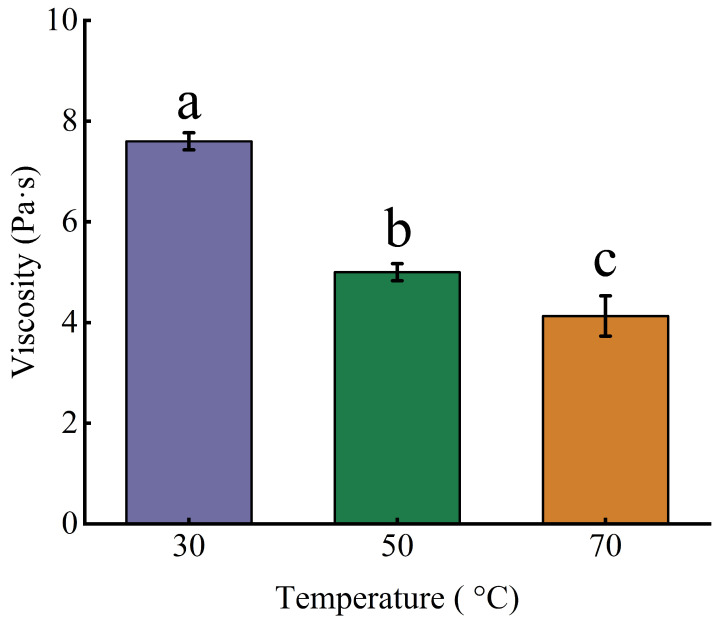
Viscosity of GJP at 30, 50, and 70 °C. The different letters indicate significant differences between groups (*p* < 0.05).

As shown in Figure 5, the viscosity of GJP progressively decreased with increasing temperature. As the temperature increased from 30 °C to 50 °C and 70 °C, the viscosity of GJP dropped from 7.60 ± 0.17 to 5.00 ± 0.17 and 4.13 ± 0.43 Pa·s, respectively. Similarly, Zhang et al. [48] reported that konjac glucomannan exhibited a viscosity of 3.4 Pa·s at 20 °C, and 0.7 Pa·s at 90 °C. Thus, GJP showed higher viscosity and stability than konjac glucomannan. Therefore, GJP obtained through optimized ultrasound-assisted extraction possesses high viscosity and thermal stability in a wide temperature range (30–70 °C).

### 3.7. Esterification Degree of GJP

The esterification degree of GJP was 2.60% ± 0.24% (Table 5), falling into the category of low-ester pectin [49]. This phenomenon was attributed to the cavitation effects of ultrasound, which can disrupt ester bonds in polysaccharides, reducing the GJP esterification degree [50]. Bai et al. [51] also reported that ultrasonic extraction yielded a lower degree of esterification (26.10%) than hot-water extraction (39.60%) for polysaccharides from sweet potato stems and leaves. Low-esterification-degree polysaccharides can form a gel in the presence of calcium ions, imparting the desired texture and stability to low-sugar or sugar-free yogurt [49,52]. Thus, low-esterification-degree GJPs can serve as valuable functional ingredients.

### 3.8. FTIR Study of GJP

**Figure 6:**
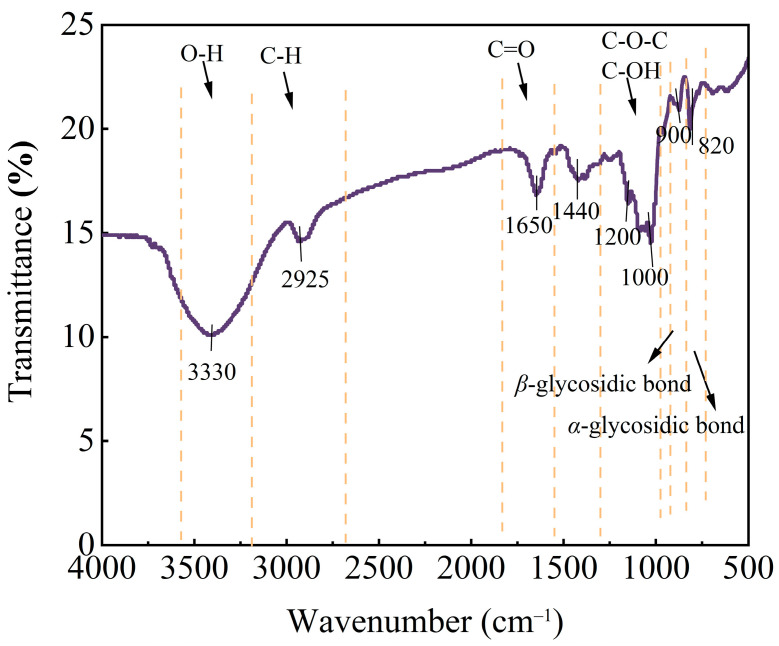
FTIR spectrum of GJP. The orange dashed line in the figure indicates the wavenumber positions of the characteristic functional groups.

The FTIR spectrum of GJP is shown in Figure 6. The broad absorption peak at 3330 cm^−1^ was observed, which was attributed to O–H stretching vibration in GJP, whereas the weak absorption peak at 2925 cm^−1^ was assigned to the C–H stretching vibration in GJP [53]. These two groups of peaks are typical absorption features of polysaccharides [54,55,56]. The absorption peak at 1650 cm^−1^ for the C=O stretching vibration indicates the presence of carbonyl groups (e.g., aldehyde or ketone groups) in GJP [56]. The weak intensity of this peak implies that carbonyl groups are not the major groups in GJP. A relatively weak peak observed between 1440 and 1350 cm^−1^ was assigned to the C–H deformation vibration [57]. Absorption peaks in the 1000–1200 cm^−1^ range were attributed to the stretching vibrations of C–O–C and C–O–H bonds in the pyranose ring [58,59,60]. Previous Nuclear Magnetic Resonance (NMR) studies by Liu et al. also confirmed this [1]. The peaks at 900 and 820 cm^−1^ suggested the possible presence of *β*-glycosidic bonds and *α*-glycosidic bonds in GJP, respectively [61].

### 3.9. XRD of GJP

**Figure 7:**
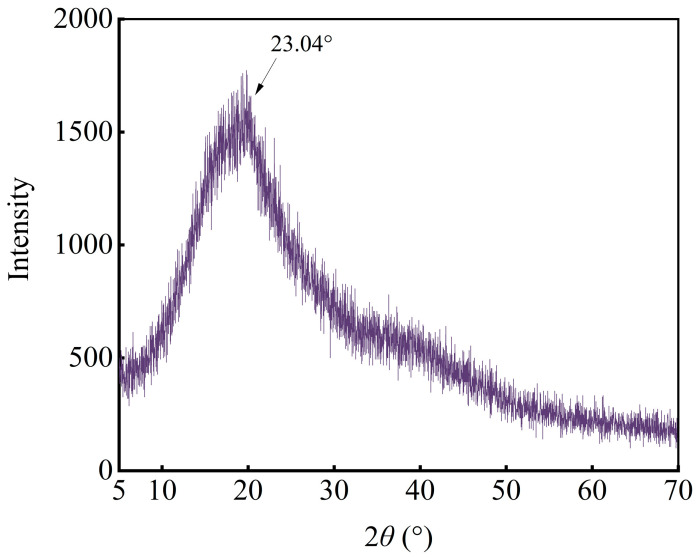
XRD spectrum of GJP at 5–70°.

The XRD pattern of GJP exhibits broad diffuse reflection peaks in the 5–70° range, with the diffraction peak observed at 2*θ* = 23.04° (Figure 7). The data indicate that GJP is a semi-crystalline polymer containing both crystalline and amorphous structures, with the amorphous phase constituting the predominant portion [48]. Intermolecular bonds in amorphous regions are typically weaker than those in crystalline regions, resulting in polysaccharides with a high proportion of amorphous regions exhibiting superior solubility [62]. This finding aligns with the high solubility (80.06% ± 2.45%) of GJP reported in Section 3.5.

### 3.10. Comparative Study on GJP Extraction: Ultrasound-Assisted Extraction vs. Hot-Water Extraction and Enzyme Extraction

To evaluate the efficacy of ultrasound-assisted extraction and determine its optimal parameters, the present study compared the current results with those of our previous study [1] (Table 6) and those for conventional hot-water extraction [13]. The optimized ultrasound-assisted extraction achieved a GJP extraction yield of 76.11% and a purity of 79.9% using water as the solvent. The extraction time was 62 min, and the energy consumption was 0.18 kW·h. In contrast, the traditional hot-water extraction method, also using water as the solvent, required 60 min of extraction and consumed 0.50 kW·h of energy. Although it yielded a slightly higher purity (83.40%), the overall extraction yield was only 16.70%. This indicates that the ultrasound-assisted method is more efficient in terms of yield and energy while yielding GJP with only slightly lower purity. Additionally, Liu et al. [1] employed the cellulase-based method for GJP extraction. The purity of GJP (49.58%) was lower than that obtained in the present study, whereas the extraction time (92 min) and energy consumption (0.75 kW·h) were higher. Furthermore, the cellulase and hot-water methods are more expensive and less environmentally friendly than the proposed ultrasound-assisted method. In summary, ultrasound-assisted extraction overcomes the issues of low yield in traditional hot-water extraction, high energy consumption in enzymatic extraction, low efficiency, high cost and environmental pollution. This makes the employed extraction process more sustainable, economical and environmentally friendly.

## 4. Conclusions and Outlook

### 4.1. Conclusions

Through a systematic approach involving single-factor, PB, steepest-climb and BB experiments, we optimized the ultrasound-assisted extraction of GJPs and established the optimal parameters as a 200 mL/g liquid-solid ratio, 62 min ultrasonic time, 51 °C ultrasonic temperature, 180 W ultrasonic power, and a single number of extractions. Operating without chemical reagents, ultrasound-assisted GJP extraction delivered a high extraction yield of 76.11% and purity of 79.89% while consuming a remarkably low energy of 0.18 kW·h. Therefore, the optimized ultrasound-assisted extraction process is a green and efficient method. Furthermore, the prepared GJP exhibited high solubility, a low degree of esterification and semi-crystalline structure. These properties enable GJP to act as an effective thickener and stabilizer in dairy products. The purity of GJP was determined to be 79.89%, meeting the standard for food additives. Overall, this study proposes a green process for the extraction of high-purity GJP.

### 4.2. Outlook

At the same time, this study has limitations, as the optimization was single-objective, focusing solely on maximizing the extraction yield. The biological activity of functional polysaccharides, such as antioxidant, immunomodulatory and prebiotic effects, is typically closely linked to their structural characteristics, such as molecular weight, monosaccharide composition and glycosidic linkage patterns, which may be affected by extraction parameters. Therefore, in future research, multi-objective optimization should be performed to balance yield with specific biological activity or target functional properties. Fundamental component analysis and purification of the target product should also be conducted. Additionally, more detailed structural characterization is required. Molecular weight, monosaccharide composition, thermogravimetry, Congo red staining, circular dichroism, methylation analysis and NMR spectroscopy should be employed to conduct preliminary analyses of the primary and higher-order structures of GJP. Additionally, in-depth functional activity analysis should be performed to investigate structure–activity relations. This would provide a reference for the in-depth development of *G. japonica* var. *delavayi* seed resources and the utilization of GJP in the design of medical foods and health supplements.

## Figures and Tables

**Table 1:** Single-factor experiment.

Group	Liquid-Solid Ratio, *X*_1_ (mL/g)	Ultrasonic Time, *X*_2_ (Min)	Ultrasonic Temperature, *X*_3_ (°C)	Ultrasonic Power, *X*_4_ (W)	Number of Extractions, *X*_5_ (Times)
1–5	100/150/200 /250/300	60	50	180	1
6–10	200	20/40/60 /80/100	50	180	1
11–15	200	60	30/40/50 /60/70	180	1
16–20	200	60	50	180/240/300 /360/420	1
21–25	200	60	50	180	1/2/3/4/5

**Table 2:** The factors and levels of Box–Behnken optimization.

Factor	Level		
	−1	0	1
Liquid-solid ratio, *X*_1_ (mL/g)	175	200	225
Ultrasonic time, *X*_2_ (min)	55	60	65
Ultrasonic temperature, *X*_3_ (°C)	45	50	55

**Table 3:** ANOVA results of Plackett–Burman experiment.

Source	Coefficient Estimate	Degree of Freedom	*F*-Value	*p*-Value
Model	468.21	5	15.84	0.0021
Liquid-solid ratio, *X*_1_ (mL/g)	70.61	1	11.94	0.0135
Ultrasonic time, *X*_2_ (min)	98.10	1	16.59	0.0066
Ultrasonic temperature, *X*_3_ (°C)	166.39	1	28.14	0.0018
Ultrasonic power, *X*_4_ (W)	50.96	1	8.62	0.0261
Number of extractions, *X*_5_ (times)	82.15	1	13.89	0.0198

**Table 4:** ANOVA analysis of Box–Behnken experiments.

Source	Sum of Squares	*DF*	Mean Square	*F*-Value	*p*-Value	Significance
Model	101.94	9	11.33	49.26	0.0001	**
*X*_1_	1.90	1	1.90	8.27	0.0238	*
*X*_2_	15.76	1	15.76	68.55	0.0001	**
*X*_3_	17.08	1	17.08	74.28	0.0001	**
*X*_1_ *X*_2_	0.0030	1	0.0030	0.0132	0.9119	
*X*_1_ *X*_3_	3.01	1	3.01	13.09	0.0085	**
*X*_2_ *X*_3_	4.88	1	4.88	21.24	0.0025	**
*X*_1_^2^	12.33	1	12.33	53.6	0.0002	**
*X*_2_^2^	19.08	1	19.08	82.95	0.0001	**
*X*_3_^2^	21.76	1	21.76	94.64	0.0001	**
Residual	1.61	7	0.23			
Lack-of-fit	0.8894	3	0.2965	1.65	0.3136	
Pure error	0.7203	4	0.1801			
Cor total	103.55	16				
*R*^2^ = 0.9845		*R*^2^_adj_ = 0.9645		C.V. (%) = 0.6630		

Note: Significant value at * < 0.05, ** < 0.01. *X*_1_ is the liquid-solid ratio, mg/mL; *X*_2_ is the ultrasonic time, min; *X*_3_ is the ultrasonic temperature, °C. *DF*, degree of freedom.

**Table 5:** Purity, protein content, solubility, and esterification degree of GJP.

Properties	Purity (%)	Protein Content (%)	Solubility (%)	Esterification Degree (%)
Values	79.89 ± 1.56	0.18 ± 0.01	80.06 ± 2.45	2.60 ± 0.24

**Table 6:** Comparison of extraction solvent, extraction time, energy consumption, extraction yield and purify of polysaccharide of different extraction methods.

Extraction Method	Extraction Solvent	Extraction Time, Min	Energy Consumption, kW·h	Yield, %	Purity, %
Ultrasound extraction	H_2_O	62	0.18	76.11	79.89
Hot-water extraction [13]	H_2_O	60	0.50	16.70	83.40
Enzymic extraction [1]	Na_2_HPO_4_/NaH_2_PO_4_	90	0.75	82.2	49.6

## Data Availability

The original contributions presented in the study are included in the article/Supplementary Materials. Further inquiries can be directed to the corresponding authors.

## Supplementary Materials

The following supporting information can be downloaded at: https://www.mdpi.com/article/10.3390/foods15071139/s1, Table S1: The factors and levels of Plackett-Burman experiment; Table S2: The factors and levels of steepest climb experiment; Table S3: Design and results of Plackett-Burman experiment; Table S4: Design and results of steepest climb experiment; Table S5: Design of Box-Behnken experiment and the extraction yield of GJP; Table S6: The actual and predicted values for the GJP extraction yield; Supplementary Text S1: MATLAB slicing function programming is employed to generate 4D plots where extraction rates are represented as independent variable functions.

## Abbreviations

The following abbreviations are used in this manuscript:

GJP	*Gleditsia japonica* var. *delavayi* polysaccharides
PB	Plackett–Burman
BB	Box–Behnken
ED	Esterification degree
FTIR	Fourier transform infrared spectrum
ANOVA	Analysis of variance
XRD	X-ray Diffraction
NMR	Nuclear magnetic resonance
