# Supplementary material for: Green Extraction of Polysaccharides from Gleditsia japonica Var. delavayi Seeds: Optimization and Physicochemical Properties
Source: Foods. 2026 Mar 26;15(7):1139. doi: 10.3390/foods15071139 (PMC13074164; doi:10.3390/foods15071139)
Supplement: Supplementary file 1 [file foods-15-01139-s001.zip › Supplementary Tables S1-S6.pdf]

Green Extraction of Polysaccharides of *Gleditsia Japonica* var. *Delavayi* Seed: Optimization and Characterization

Hongying Li [a, †], Chengyan Pi [a, †], Xiaowei Peng [a], Boxiao Wu [a, b], Changwei Cao [a, b], Huan Kan [a, b], Yun Liu [a, b, *], Fang Li [a, b, *]

[a] *College of Biological Science and Food Engineering, Southwest Forestry University, Kunming 650224, China*

[b] *Forest Resources Exploitation and Utilization Engineering Research Center for Grand Health of Yunnan Provincial Universities, Southwest Forestry University, Kunming 650224, China*

* Corresponding author: Fang Li (lifang@swfu.edu.cn)

Yun Liu (liuyun@swfu.edu.cn)

[†] These authors contributed equally to this work.

Supplementary Table S1 The factors and levels of Plackett-Burman experiment.

**Factor**	**Minimum value (−1)**	**Maximum value (1)**
Liquid-solid ratio, X_1 (mL/g)	150	250
Ultrasonic time, X_2 (min)	40	80
Ultrasonic temperature, X_3 (°C)	30	60
Ultrasonic power, X_4 (W)	180	420
Number of extractions, X_5 (times)	1	3

Supplementary Table S2 The factors and levels of steepest climb experiment.

**Runs**	**Liquid-solid ratio, X_1 (mL/g)**	**Ultrasonic time, X_2 (min)**	**Ultrasonic temperature, X_3 (°C)**
1	150	50	40
2	175	55	45
3	200	60	50
4	225	65	55
5	250	70	60

Supplementary Table S3 Design and results of Plackett-Burman experiment.

**Runs**	**Liquid-solid ratio, X_1 (mL/g)**	**Ultrasonic time, X_2 (min)**	**Ultrasonic temperature, X_3 (°C)**	**Ultrasonic power, X_4 (W)**	**Number of extractions, X_5 (times)**	**Extraction yield, (%)**
1	250	80	30	420	1	**72.25 ± 0.56**
2	250	40	30	180	3	**68.64 ± 2.42**
3	250	80	60	180	3	**80.80 ± 0.71**
4	150	40	30	180	1	**59.94 ± 1.14**

5	150	80	30	420	3	**72.54 ± 4.66**
6	250	80	30	180	1	**70.34 ± 0.34**
7	150	40	60	180	1	**62.80 ± 2.44**
8	150	80	60	180	3	**80.09 ± 2.19**
9	250	40	60	420	3	**79.67 ± 0.78**
10	150	40	30	420	3	**68.93 ± 4.34**
11	150	80	60	420	1	**76.12 ± 0.32**
12	250	40	60	420	1	**77.84 ± 0.56**

Supplementary Table S4 Design and results of steepest climb experiment.

Runs	Liquid-solid ratio, X_1 (mL/g)	Ultrasonic time, X_2 (min)	Ultrasonic temperature, X_3 (°C)	Extraction yield (%)
1	150	50	40	55.58 ± 1.85
2	175	55	45	75.11 ± 1.15
3	200	60	50	76.11 ± 0.07
4	225	65	55	72.23 ± 0.29
5	250	70	60	67.89 ± 0.17

Supplementary Table S5 Design of Box-Behnken experiment and the extraction yield of GJP.

Runs	Liquid-solid ratio, X_1 (mL/g)	Ultrasonic time, X_2 (min)	Ultrasonic temperature, X_3 (°C)	Predicted extraction yield (%)	Actual extraction yield (%)
1	225	60	45	69.4	69.80 ± 1.85
2	200	60	50	75.2	74.62 ± 0.29
3	225	65	50	73.2	72.92 ± 0.60
4	225	55	50	70.5	70.41 ± 2.90

5	175	55	50	69.4	69.79 ± 0.31
6	200	60	50	75.2	75.47 ± 0.67
7	200	60	50	75.2	75.71 ± 0.52
8	200	55	55	72.0	72.01 ± 0.47
9	200	60	50	75.2	75.28 ± 0.46
10	200	60	50	75.2	74.94 ± 1.09
11	175	60	55	71.3	70.92 ± 0.28
12	175	65	50	72.3	72.43 ± 1.13
13	175	60	45	70.1	70.11 ± 1.18
14	200	65	45	71.9	71.77 ± 0.67
15	200	55	45	66.8	66.52 ± 0.81
16	225	60	55	74.0	74.04 ± 0.52
17	200	65	55	72.6	72.91 ± 1.03

Supplementary Table S6 The actual and predicted values for the GJP extraction yield.

	**Liquid-solid ratio, X_1 (mL/g)**	**Ultrasonic time, X_2 (min)**	**Ultrasonic temperature, X_3 (°C)**	**Extraction yield (%)**
Actual value	200	62	51	76.11 ± 0.43
Predicted value	200	61.4	50.5	75.5
