# Supplementary material for: Green Extraction of Polysaccharides from Gleditsia japonica Var. delavayi Seeds: Optimization and Physicochemical Properties
Source: Foods. 2026 Mar 26;15(7):1139. doi: 10.3390/foods15071139 (PMC13074164; doi:10.3390/foods15071139)
Supplement: Supplementary file 1 [file foods-15-01139-s001.zip › Supplementary Text S1.pdf]

Green Extraction of Polysaccharides of *Gleditsia Japonica* var. *Delavayi* Seed: Optimization and Characterization

Hongying Li [a, †], Chengyan Pi [a, †], Xiaowei Peng [a], Boxiao Wu [a, b], Changwei Cao [a, b], Huan Kan [a, b], Yun Liu [a, b, *], Fang Li [a, b, *]

[a] *College of Biological Science and Food Engineering, Southwest Forestry University, Kunming 650224, China*

[b] *Forest Resources Exploitation and Utilization Engineering Research Center for Grand Health of Yunnan Provincial Universities, Southwest Forestry University, Kunming 650224, China*

* Corresponding author: Fang Li (lifang@swfu.edu.cn)

Yun Liu (liuyun@swfu.edu.cn)

† These authors contributed equally to this work.

Supplementary Text S1 MATLAB slicing function programming is employed to generate 4D plots where extraction rates are represented as independent variable functions.

```
>> % 1. Prepare training data (directly from Excel)
% Input data: Liquid-to-material ratio(mg/mL), Ultrasonic time(min), Ultrasonic temperature(°C)
inputs = [
    225, 200, 225, 225, 175, 200, 200, 200, 200, 200, 175, 175, 175, 200, 200, 225, 200;   %
Liquid-to-material ratio
    60,   60,   65,   55,   55,   60,   60,   55,   60,   60,   60,   65,   60,   65,   55,   60,
65;    % Ultrasonic time
    45,   50,   50,   50,   50,   50,   50,   55,   50,   50,   55,   50,   45,   45,   45,   55,
55     % Ultrasonic temperature
];

% Output data (yield) - directly from Excel
targets = [
    69.8, 74.6, 72.9, 70.4, 69.8, 75.5, 75.7, 72.0, 75.3, 74.9, 70.9, 72.4, 70.1, 71.8, 66.5, 74.0, 72.9
];

% Set global font to Times New Roman
set(0, 'DefaultAxesFontName', 'Times New Roman');
set(0, 'DefaultTextFontName', 'Times New Roman');
set(0, 'DefaultUicontrolFontName', 'Times New Roman');
set(0, 'DefaultUitableFontName', 'Times New Roman');
set(0, 'DefaultUipanelFontName', 'Times New Roman');

% 2. Create and train neural network
% Determine input ranges for subsequent plotting
x_range = [min(inputs(1,:)), max(inputs(1,:))];   % Liquid-to-material ratio: 175-225
y_range = [min(inputs(2,:)), max(inputs(2,:))];   % Ultrasonic time: 55-65
z_range = [min(inputs(3,:)), max(inputs(3,:))];   % Ultrasonic temperature: 45-55

% Create neural network
try
    % Try using newfit (older MATLAB versions)
    net = newfit(inputs, targets, 10);   % 10 hidden neurons
    net.trainParam.epochs = 1000;
    net.trainParam.goal = 1e-5;
    net = train(net, inputs, targets);
```

```
catch
    % If newfit is not available, use feedforwardnet (newer MATLAB versions)
    net = feedforwardnet(10);
    net.trainParam.epochs = 1000;
    net.trainParam.goal = 1e-5;
    net = train(net, inputs, targets);
end

% 3. Generate denser grid for plotting
x_plot = linspace(x_range(1), x_range(2), 30);
y_plot = linspace(y_range(1), y_range(2), 30);
z_plot = linspace(z_range(1), z_range(2), 30);
[X_plot, Y_plot, Z_plot] = meshgrid(x_plot, y_plot, z_plot);

% 4. Predict response surface
P_plot = [X_plot(:)'; Y_plot(:)'; Z_plot(:)'];
Q_plot = sim(net, P_plot);
V_plot = reshape(Q_plot, size(X_plot));

% 5. Plot slice diagram
figure('Position', [100, 100, 1400, 900]);

% Set slice positions based on actual data range
Xslice = [180, 210];    % Liquid-to-material ratio cross-sections
Yslice = [58, 62];          % Ultrasonic time cross-sections
Zslice = [48, 52];          % Ultrasonic temperature cross-sections

% Plot slices
h_slice = slice(X_plot, Y_plot, Z_plot, V_plot, Xslice, Yslice, Zslice);

% Set slice transparency
for i = 1:length(h_slice)
    set(h_slice(i), 'EdgeColor', 'none', 'FaceAlpha', 0.8);
end

% Set graphic properties
colormap(jet);
h_colorbar = colorbar;
set(h_colorbar, 'FontName', 'Times New Roman', 'FontSize', 12);
caxis([66, 76]); % Set color range to highlight yield variation

xlabel('Liquid-to-material ratio (mg/mL)', 'FontSize', 14, 'FontWeight', 'bold');
ylabel('Ultrasonic time (min)', 'FontSize', 14, 'FontWeight', 'bold');
zlabel('Ultrasonic temperature (°C)', 'FontSize', 14, 'FontWeight', 'bold');
```

```
title('Three-factor Response Surface Analysis of Ultrasonic Extraction - Yield Distribution', 'FontSize', 16, 'FontWeight', 'bold');

% Add grid and lighting effects
grid on;
lighting gouraud;
shading interp;

% 6. Mark all experimental points
hold on;
% Mark all experimental points with the same color
plot3(inputs(1,:), inputs(2,:), inputs(3,:), ...
    'ro', 'MarkerSize', 10, 'MarkerFaceColor', 'r', 'DisplayName', 'Experimental points');

% Highlight center point (200, 60, 50)
center_point = find(inputs(1,:)==200 & inputs(2,:)==60 & inputs(3,:)==50);
plot3(inputs(1,center_point), inputs(2,center_point), inputs(3,center_point), ...
    'gs', 'MarkerSize', 12, 'MarkerFaceColor', 'g', 'DisplayName', 'Center point (200,60,50)');

h_legend = legend('Location', 'northeast', 'FontSize', 12);
set(h_legend, 'FontName', 'Times New Roman');

% Set axes font properties
set(gca, 'FontName', 'Times New Roman', 'FontSize', 12);

% 7. Find and mark optimal condition
[max_val, max_idx] = max(Q_plot);
optimal_x = P_plot(1, max_idx);
optimal_y = P_plot(2, max_idx);
optimal_z = P_plot(3, max_idx);

fprintf('Predicted optimal conditions:\n');
fprintf('Liquid-to-material ratio: %.1f mg/mL\n', optimal_x);
fprintf('Ultrasonic time: %.1f min\n', optimal_y);
fprintf('Ultrasonic temperature: %.1f °C\n', optimal_z);
fprintf('Predicted yield: %.2f%%\n', max_val);

% Mark optimal condition point
plot3(optimal_x, optimal_y, optimal_z, 'pentagram', ...
    'MarkerSize', 18, 'MarkerFaceColor', 'yellow', ...
    'MarkerEdgeColor', 'black', 'LineWidth', 2, ...
    'DisplayName', sprintf('Predicted optimum (%.1f%%)', max_val));

% 8. Calculate and display model performance
```

```
predictions = sim(net, inputs);
mse_value = mean((predictions - targets).^2);
rmse_value = sqrt(mse_value);
r2 = 1 - sum((targets - predictions).^2) / sum((targets - mean(targets)).^2);

fprintf('\nNeural network model performance:\n');
fprintf('Mean Square Error (MSE): %.4f\n', mse_value);
fprintf('Root Mean Square Error (RMSE): %.4f\n', rmse_value);
fprintf('Coefficient of Determination (R²): %.4f\n', r2);

% 9. Plot predicted vs actual values comparison
figure('Position', [100, 100, 800, 600]);
plot(targets, predictions, 'bo', 'MarkerSize', 8, 'MarkerFaceColor', 'b');
hold on;
plot([min(targets), max(targets)], [min(targets), max(targets)], 'r--', 'LineWidth', 2);
xlabel('Actual yield (%)', 'FontSize', 12, 'FontWeight', 'bold');
ylabel('Predicted yield (%)', 'FontSize', 12, 'FontWeight', 'bold');
title('Neural Network Prediction Performance Verification', 'FontSize', 14, 'FontWeight', 'bold');
h_legend2 = legend('Experimental data', 'Ideal fit line', 'Location', 'best');
set(h_legend2, 'FontName', 'Times New Roman');
grid on;
set(gca, 'FontName', 'Times New Roman', 'FontSize', 11);

% Add R² value to the graph
text(min(targets)+1, max(predictions)-1, ...
    sprintf('R² = %.4f', r2), 'FontSize', 12, 'BackgroundColor', 'white', 'FontName', 'Times New Roman');

% 10. Plot residual graph
figure('Position', [100, 100, 800, 600]);
residuals = predictions - targets;
plot(predictions, residuals, 'bo', 'MarkerSize', 8, 'MarkerFaceColor', 'b');
hold on;
plot([min(predictions), max(predictions)], [0, 0], 'r-', 'LineWidth', 2);
xlabel('Predicted yield (%)', 'FontSize', 12, 'FontWeight', 'bold');
ylabel('Residuals', 'FontSize', 12, 'FontWeight', 'bold');
title('Residual Analysis', 'FontSize', 14, 'FontWeight', 'bold');
grid on;
set(gca, 'FontName', 'Times New Roman', 'FontSize', 11);

% 11. Plot relationships between factors and yield (marginal effects)
figure('Position', [100, 100, 1200, 400]);

% Liquid-to-material ratio vs yield
```

```
subplot(1,3,1);
scatter(inputs(1,:), targets, 60, 'filled', 'b');
xlabel('Liquid-to-material ratio (mg/mL)', 'FontSize', 12, 'FontWeight', 'bold');
ylabel('Yield (%)', 'FontSize', 12, 'FontWeight', 'bold');
title('Liquid-to-material ratio vs Yield', 'FontSize', 12, 'FontWeight', 'bold');
grid on;
set(gca, 'FontName', 'Times New Roman', 'FontSize', 11);

% Ultrasonic time vs yield
subplot(1,3,2);
scatter(inputs(2,:), targets, 60, 'filled', 'b');
xlabel('Ultrasonic time (min)', 'FontSize', 12, 'FontWeight', 'bold');
ylabel('Yield (%)', 'FontSize', 12, 'FontWeight', 'bold');
title('Ultrasonic time vs Yield', 'FontSize', 12, 'FontWeight', 'bold');
grid on;
set(gca, 'FontName', 'Times New Roman', 'FontSize', 11);

% Ultrasonic temperature vs yield
subplot(1,3,3);
scatter(inputs(3,:), targets, 60, 'filled', 'b');
xlabel('Ultrasonic temperature (°C)', 'FontSize', 12, 'FontWeight', 'bold');
ylabel('Yield (%)', 'FontSize', 12, 'FontWeight', 'bold');
title('Ultrasonic temperature vs Yield', 'FontSize', 12, 'FontWeight', 'bold');
grid on;
set(gca, 'FontName', 'Times New Roman', 'FontSize', 11);

% 12. Plot 2D contour plots of different cross-sections
figure('Position', [100, 100, 1500, 400]);

% Fix ultrasonic temperature at 50°C
subplot(1,3,1);
temp_fixed = 50;
temp_idx = find(abs(z_plot - temp_fixed) == min(abs(z_plot - temp_fixed)), 1);
contourf(X_plot(:,:,temp_idx), Y_plot(:,:,temp_idx), V_plot(:,:,temp_idx), 20, 'LineStyle', 'none');
h_cb1 = colorbar;
set(h_cb1, 'FontName', 'Times New Roman', 'FontSize', 10);
xlabel('Liquid-to-material ratio (mg/mL)', 'FontSize', 12, 'FontWeight', 'bold');
ylabel('Ultrasonic time (min)', 'FontSize', 12, 'FontWeight', 'bold');
title(sprintf('Yield at ultrasonic temperature %d°C', temp_fixed), 'FontSize', 12, 'FontWeight', 'bold');
set(gca, 'FontName', 'Times New Roman', 'FontSize', 11);

% Fix ultrasonic time at 60min
subplot(1,3,2);
time_fixed = 60;
```

```
time_idx = find(abs(y_plot - time_fixed) == min(abs(y_plot - time_fixed)), 1);
contourf(squeeze(X_plot(time_idx,:,:)), squeeze(Z_plot(time_idx,:,:)), squeeze(V_plot(time_idx,:,:)), 20, 'LineStyle', 'none');
h_cb2 = colorbar;
set(h_cb2, 'FontName', 'Times New Roman', 'FontSize', 10);
xlabel('Liquid-to-material ratio (mg/mL)', 'FontSize', 12, 'FontWeight', 'bold');
ylabel('Ultrasonic temperature (°C)', 'FontSize', 12, 'FontWeight', 'bold');
title(sprintf('Yield at ultrasonic time %dmin', time_fixed), 'FontSize', 12, 'FontWeight', 'bold');
set(gca, 'FontName', 'Times New Roman', 'FontSize', 11);

% Fix liquid-to-material ratio at 200mg/mL
subplot(1,3,3);
ratio_fixed = 200;
ratio_idx = find(abs(x_plot - ratio_fixed) == min(abs(x_plot - ratio_fixed)), 1);
contourf(squeeze(Y_plot(:,ratio_idx,:)), squeeze(Z_plot(:,ratio_idx,:)), squeeze(V_plot(:,ratio_idx,:)), 20, 'LineStyle', 'none');
h_cb3 = colorbar;
set(h_cb3, 'FontName', 'Times New Roman', 'FontSize', 10);
xlabel('Ultrasonic time (min)', 'FontSize', 12, 'FontWeight', 'bold');
ylabel('Ultrasonic temperature (°C)', 'FontSize', 12, 'FontWeight', 'bold');
title(sprintf('Yield at liquid-to-material ratio %dmg/mL', ratio_fixed), 'FontSize', 12, 'FontWeight', 'bold');
set(gca, 'FontName', 'Times New Roman', 'FontSize', 11);

% 13. Display experimental design information
fprintf('\nExperimental Design Information:\n');
fprintf('Number of experiments: %d\n', length(targets));
fprintf('Liquid-to-material ratio range: %d-%d mg/mL\n', x_range(1), x_range(2));
fprintf('Ultrasonic time range: %d-%d min\n', y_range(1), y_range(2));
fprintf('Ultrasonic temperature range: %d-%d °C\n', z_range(1), z_range(2));
fprintf('Yield range: %.1f-%.1f%%\n', min(targets), max(targets));

% Reset default fonts to system default (optional)
% set(0, 'DefaultAxesFontName', 'remove');
% set(0, 'DefaultTextFontName', 'remove');
```
